# (*E*)-4-[2-(4-Eth­oxy­phen­yl)ethen­yl]-1-methyl­pyridinium 4-bromo­benzene­sulfonate methanol hemisolvate^1^

**DOI:** 10.1107/S1600536810050944

**Published:** 2010-12-11

**Authors:** Hoong Kun Fun, Suchada Chantrapromma, Patcharaporn Jansrisewangwong

**Affiliations:** a X-ray Crystallography Unit, School of Physics, Universiti Sains Malaysia, 11800 USM, Penang, Malaysia; b Crystal Materials Research Unit, Department of Chemistry, Faculty of Science, Prince of Songkla University, Hat-Yai, Songkhla 90112, Thailand

## Abstract

In the title compound, C_16_H_18_NO^+^·C_6_H_4_BrO_3_S^−^·0.5CH_3_OH, the cation exists in the *E* configuration and the whole mol­ecule of the cation, except for the O atom of the eth­oxy group, is disordered with a site-occupancy ratio of 0.695 (5):0.305 (5). The cation is disordered in such a way that the ethenyl units of the major and minor components are related by 180° around the long mol­ecular axis. In the major component, the cation is almost planar, the dihedral angle between the pyridinium and benzene rings being 0.8 (3)°, whereas in the minor component, the dihedral angle between the two aromatic rings is 4.2 (6)°. In the crystal, the cations are stacked in an anti­parallel manner along the *a* axis, while the anions and methanol mol­ecules are linked through O—H⋯O hydrogen bonds and Br⋯O short contacts [3.0248 (13) Å] into a tape along the same direction. The three components are further linked by weak C—H⋯O, C—H⋯Br and C—H⋯π inter­actions.

## Related literature

For bond-length data, see: Allen *et al.* (1987 ▶). For background to non-linear optical materials research, see: Cheng, Tam, Marder *et al.* (1991 ▶); Cheng, Tam, Stevenson *et al.* (1991 ▶); Ogawa *et al.* (2008 ▶); Ruanwas *et al.* (2010 ▶); Yang *et al.* (2007 ▶). For related structures, see: Chantrapromma *et al.* (2006 ▶); Chantrapromma, Chanawanno & Fun (2009 ▶); Chantra­promma, Jansrisewangwong *et al.* (2009 ▶); Fun *et al.* (2009 ▶). For the stability of the temperature controller used in the data collection, see: Cosier & Glazer (1986 ▶).
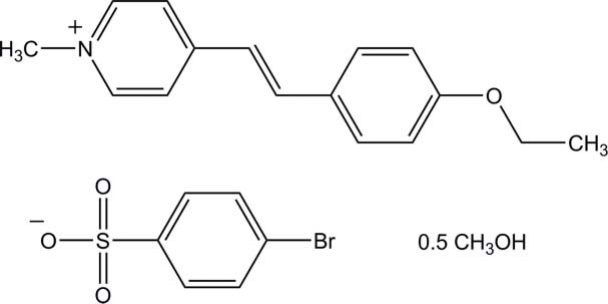


## Experimental

### 

#### Crystal data

2C_16_H_18_NO^+^·2C_6_H_4_BrO_3_S^−^·CH_4_O
*M* *_r_* = 984.79
Triclinic, 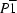

*a* = 9.9270 (4) Å
*b* = 9.9813 (4) Å
*c* = 11.5293 (4) Å
α = 75.703 (2)°
β = 76.965 (2)°
γ = 88.395 (2)°
*V* = 1078.00 (7) Å^3^
*Z* = 1
Mo *K*α radiation
μ = 2.04 mm^−1^
*T* = 100 K
0.58 × 0.41 × 0.17 mm

#### Data collection

Bruker APEXII CCD area-detector diffractometer
Absorption correction: multi-scan (*SADABS*; Bruker, 2005 ▶) *T*_min_ = 0.383, *T*_max_ = 0.721
24757 measured reflections
6214 independent reflections
5389 reflections with *I* > 2σ(*I*)
*R*_int_ = 0.030

#### Refinement

*R*[*F* ^2^ > 2σ(*F* ^2^)] = 0.033
*wR*(*F* ^2^) = 0.086
*S* = 1.03
6214 reflections
354 parameters
6 restraints
H-atom parameters constrained
Δρ_max_ = 1.47 e Å^−3^
Δρ_min_ = −0.54 e Å^−3^

### 

Data collection: *APEX2* (Bruker, 2005 ▶); cell refinement: *SAINT* (Bruker, 2005 ▶); data reduction: *SAINT*; program(s) used to solve structure: *SHELXTL* (Sheldrick, 2008 ▶); program(s) used to refine structure: *SHELXTL*; molecular graphics: *SHELXTL*; software used to prepare material for publication: *SHELXTL* and *PLATON* (Spek, 2009 ▶).

## Supplementary Material

Crystal structure: contains datablocks global, I. DOI: 10.1107/S1600536810050944/is2636sup1.cif

Structure factors: contains datablocks I. DOI: 10.1107/S1600536810050944/is2636Isup2.hkl

Additional supplementary materials:  crystallographic information; 3D view; checkCIF report

## Figures and Tables

**Table 1:** Hydrogen-bond geometry (Å, °) *Cg*1, *Cg*2, *Cg*3, *Cg*4 and *Cg*5 are the centroids of the N1*A*/C1*A*–C5*A*, C8*A*–C13*A*, N1*B*/C1*B*–C5*B*, C8*B*–C13*B* and C17–C22 rings, respectively.

*D*—H⋯*A*	*D*—H	H⋯*A*	*D*⋯*A*	*D*—H⋯*A*
O5—H5⋯O3^i^	0.82	1.92	2.657 (4)	149
C1*A*—H1*AA*⋯O2^ii^	0.93	2.42	3.334 (4)	168
C2*A*—H2*AA*⋯O2^iii^	0.93	2.38	3.237 (5)	153
C14*A*—H14*A*⋯O4^iv^	0.96	2.36	3.180 (6)	143
C14*A*—H14*B*⋯O4^iii^	0.96	2.41	3.343 (6)	163
C19—H19*A*⋯O5^v^	0.93	2.53	3.385 (4)	153
C21—H21*A*⋯O2^vi^	0.93	2.58	3.311 (2)	135
C23—H23*C*⋯Br1	0.96	2.77	3.724 (5)	173
C14*A*—H14*C*⋯*Cg*2^ii^	0.96	2.66	3.609 (6)	172
C14*A*—H14*C*⋯*Cg*4^ii^	0.96	2.63	3.572 (8)	167
C15*A*—H15*A*⋯*Cg*1^vii^	0.97	2.84	3.639 (8)	140
C15*A*—H15*A*⋯*Cg*3^vii^	0.97	2.84	3.611 (9)	137
C14*B*—H14*D*⋯*Cg*2^ii^	0.96	2.87	3.562 (15)	129
C14*B*—H14*D*⋯*Cg*4^ii^	0.96	2.80	3.564 (16)	137
C15*B*—H15*C*⋯*Cg*1^vii^	0.97	2.81	3.65 (3)	145
C15*B*—H15*C*⋯*Cg*3^vii^	0.97	2.81	3.62 (3)	141
C15*B*—H15*D*⋯*Cg*5^viii^	0.97	2.98	3.60 (2)	123

Symmetry codes: (i) 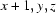; (ii) 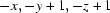; (iii) 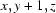; (iv) 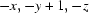; (v) 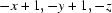; (vi) 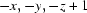; (vii) 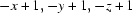; (viii) 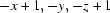.

**Fig. 1.**
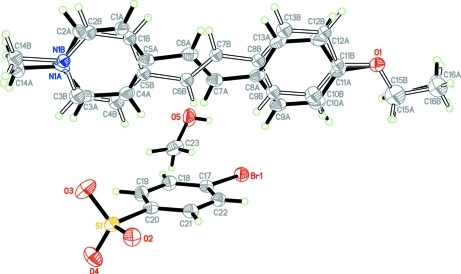
The molecular structure of the title compound, with 50% probability displacement ellipsoids and the atom-numbering scheme. Open bonds show the minor component.

**Fig. 2.**
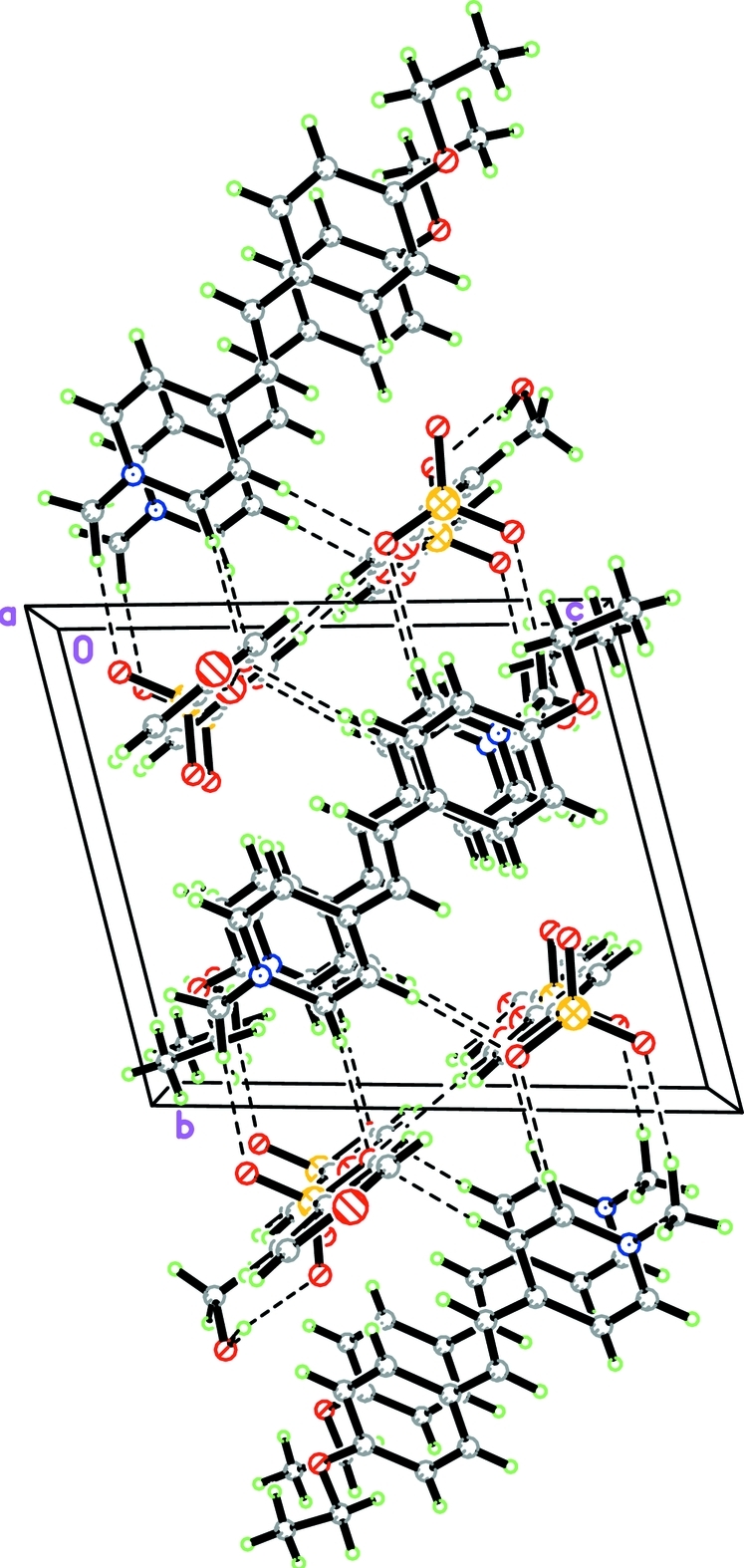
The crystal packing of the major component of the title compound viewed down the a axis. O—H···O hydrogen bonds and C—H···O weak interactions are shown as dashed lines.

## supplementary crystallographic information

### Comment

Organic crystals with extensive conjugated π systems with large hyperpolarizability which exhibit NLO properties have been reported (Ogawa *et al.*, 2008; Ruanwas *et al.*, 2010; Yang *et al.*, 2007). Styryl pyridinium derivatives are considered to be good conjugated π-systems (Cheng, Tam, Marder *et al.*, 1991; Cheng, Tam, Stevenson *et al.*, 1991). In our on-going research in searching for NLO materials (Chantrapromma *et al.*, 2006; Chantrapromma, Chanawanno & Fun, 2009; Chantrapromma, Jansrisewangwong *et al.*, 2009; Ruanwas *et al.*, 2010), the title compound (I) was synthesized. Unfortunately (I) crystallizes in the triclinic centrosymmetric space group *P*-1 and did not exhibit second-order nonlinear optical properties.

The asymmetric unit of (I) consists of one C_16_H_18_NO^+^ cation, one C_6_H_4_BrO_3_S^-^ anion and one-half of the CH_3_OH molecule. The whole molecule except the O atom of the ethoxy group (O1) of the cation is disordered over two sites with the major component *A* and the minor *B* components having refined site-occupancy ratio of 0.695 (5):0.305 (5) (Fig. 1). The cation exists in the *E* configuration with respect to the C6═C7 double bond and the torsion angle C5–C6–C7–C8 = -179.6 (2)° for major component *A* and 179.5 (6)° for minor component *B* indicating that the orientation of the ethenyl moiety in major and minor components is related by 180° rotation. In the major component *A*, the cation is planar with the dihedral angle between the pyridinium and benzene rings being 0.8 (3)°, whereas in the minor component *B*, the dihedral angle between the two aromatic rings is 4.2 (6)°. The anion is inclined to the cation with the dihedral angle between the C17–C22 benzene ring of the anion and the mean plane of the conjugated π system (C1–C13/N1) [*r.m.s* = 0.013 (2) and 0.033 (2) Å for major and minor components, respectively] of the cation being 79.73 (12) and 79.2 (2)° for major and minor components, respectively. The ethoxy group is co-planar with the attached benzene ring as indicated by the torsion angle C11A–O1–C15A–C16A = 178.8 (8)° for major component and C11B–O1–C15B–C16B = -175 (2)° for minor component. The bond lengths in (I) are in normal ranges (Allen *et al.*, 1987) and comparable to those in related structures (Chantrapromma *et al.*, 2006; Chantrapromma, Chanawanno & Fun, 2009; Chantrapromma, Jansrisewangwong *et al.*, 2009; Fun *et al.*, 2009).

In the crystal packing (Fig. 2), the cations and anions are individually arranged into chains along the *a* axis. The methanol molecules are linked to the anions by C—H···Br weak interactions and O—H···O hydrogen bonds, respectively (Table 1). The cations, anions and methanol molecules are linked together by O—H···O hydrogen bonds and C—H···O weak interactions forming sheets parallel to the *bc* plane. The crystal structure is further stabilized by C—H···π interactions (Table 1). A Br···O short contact [3.0248 (13) Å; symmetry code: 1 + *x*, *y*, *z*] was observed.

### Experimental

(*E*)-4-(4-Ethoxystyryl)-1-methylpyridinium iodide (compound A) was prepared by mixing 1:1:1 molar ratio solutions of 1,4-dimethylpyridinium iodide (2.00 g, 8.5 mmol), 4-ethoxybenzaldehyde (1.27 g, 8.5 mmol) and piperidine (0.84 ml, 8.5 mmol) in hot methanol (50 ml). The resulting solution was refluxed for 3 h under a nitrogen atmosphere. The resultant solid was filtered off and washed with diethylether to give oranged-yellow solid of compound A (2.18 g, 69%), M.p. 491-492 K. Silver (I) 4-bromobenzenesulfonate (compound B) was synthesized according to our previously reported procedure (Chantrapromma *et al.*, 2006). The title compound was synthesized by mixing a solution of compound A (0.20 g, 0.5 mmol) in hot methanol (25 ml) and a solution of compound B (0.17 g, 0.5 mmol) in hot methanol (50 ml). The mixture immediately yielded a grey precipitate of silver iodide. After stirring the mixture for 30 min, the precipitate of silver iodide was removed and the resulting solution was evaporated yielding a yellow solid of the title compound. Yellow plate-shaped single crystals of the title compound suitable for *x*-ray structure determination were recrystallized from methanol by slow evaporation of the solvent at room temperature over several days, M.p. 513-515 K.

### Refinement

All H atoms were positioned geometrically and allowed to ride on their parent atoms, with *d*(O—H) = 0.82 Å, *d*(C—H) = 0.93 Å for aromatic and CH, 0.97 Å for CH_2_ and 0.96 Å for CH_3_ atoms. The *U*_iso_ values were constrained to be 1.5*U*_eq_ of the carrier atoms for methyl H atoms and 1.2*U*_eq_ for the remaining H atoms. A rotating group model was used for the methyl groups. The highest residual electron density peak is located at 0.30 Å from H23B and the deepest hole is located at 0.68 Å from Br1. The whole cation, with the exception of the O1 atom of the ethoxy group, is disordered over two sites with a refined occupancy ratio of 0.695 (5):0.305 (5). All atoms of the minor component *B* were refined isotropically. Initially rigidity and similarity restraints were applied. After steady state has been reached, these restraints were removed and *DFIX* restraints were applied to O1—C11A, O1—C11B, O1—C15A and O1—C15B bond distances. The occupancy of the metahnol molecule was refined to 0.542 (7). In the final refinement, it was fixed to 0.5.

### Crystal data

**Table:**

2C_16_H_18_NO^+^·2C_6_H_4_BrO_3_S^−^·CH_4_O	*Z* = 1
*M**_r_* = 984.79	*F*(000) = 506
Triclinic, *P*1	*D*_x_ = 1.517 Mg m^−^^3^
Hall symbol: -P 1	Melting point = 513–515 K
*a* = 9.9270 (4) Å	Mo *K*α radiation, λ = 0.71073 Å
*b* = 9.9813 (4) Å	Cell parameters from 6214 reflections
*c* = 11.5293 (4) Å	θ = 1.9–30.0°
α = 75.703 (2)°	µ = 2.04 mm^−^^1^
β = 76.965 (2)°	*T* = 100 K
γ = 88.395 (2)°	Plate, yellow
*V* = 1078.00 (7) Å^3^	0.58 × 0.41 × 0.17 mm

### Data collection

**Table:**

Bruker APEXII CCD area-detector diffractometer	6214 independent reflections
Radiation source: sealed tube	5389 reflections with *I* > 2σ(*I*)
graphite	*R*_int_ = 0.030
φ and ω scans	θ_max_ = 30.0°, θ_min_ = 1.9°
Absorption correction: multi-scan (*SADABS*; Bruker, 2005)	*h* = −13→13
*T*_min_ = 0.383, *T*_max_ = 0.721	*k* = −14→14
24757 measured reflections	*l* = −16→16

### Refinement

**Table:**

Refinement on *F*^2^	Primary atom site location: structure-invariant direct methods
Least-squares matrix: full	Secondary atom site location: difference Fourier map
*R*[*F*^2^ > 2σ(*F*^2^)] = 0.033	Hydrogen site location: inferred from neighbouring sites
*wR*(*F*^2^) = 0.086	H-atom parameters constrained
*S* = 1.03	*w* = 1/[σ^2^(*F*_o_^2^) + (0.0421*P*)^2^ + 0.6369*P*] where *P* = (*F*_o_^2^ + 2*F*_c_^2^)/3
6214 reflections	(Δ/σ)_max_ = 0.002
354 parameters	Δρ_max_ = 1.47 e Å^−^^3^
6 restraints	Δρ_min_ = −0.54 e Å^−^^3^

### Special details

**Table:**

Experimental. The crystal was placed in the cold stream of an Oxford Cryosystems Cobra open-flow nitrogen cryostat (Cosier & Glazer, 1986) operating at 100.0 (1) K.
Geometry. All esds (except the esd in the dihedral angle between two l.s. planes) are estimated using the full covariance matrix. The cell esds are taken into account individually in the estimation of esds in distances, angles and torsion angles; correlations between esds in cell parameters are only used when they are defined by crystal symmetry. An approximate (isotropic) treatment of cell esds is used for estimating esds involving l.s. planes.
Refinement. Refinement of F^2^ against ALL reflections. The weighted R-factor wR and goodness of fit S are based on F^2^, conventional R-factors R are based on F, with F set to zero for negative F^2^. The threshold expression of F^2^ > 2sigma(F^2^) is used only for calculating R-factors(gt) etc. and is not relevant to the choice of reflections for refinement. R-factors based on F^2^ are statistically about twice as large as those based on F, and R- factors based on ALL data will be even larger.

### Fractional atomic coordinates and isotropic or equivalent isotropic displacement parameters (Å^2^)

**Table:**

	*x*	*y*	*z*	*U*_iso_*/*U*_eq_	Occ. (<1)
Br1	0.611495 (18)	0.152815 (19)	0.312050 (17)	0.02878 (6)	
S1	−0.01349 (4)	0.19135 (4)	0.23886 (4)	0.02485 (9)	
O1	0.57929 (15)	0.21814 (14)	0.88911 (12)	0.0318 (3)	
O2	−0.09844 (13)	0.10914 (14)	0.35315 (12)	0.0314 (3)	
O3	−0.04752 (16)	0.33740 (14)	0.21612 (16)	0.0415 (4)	
O4	−0.00778 (15)	0.13368 (18)	0.13490 (14)	0.0396 (3)	
O5	0.8900 (4)	0.4976 (3)	0.0154 (3)	0.0435 (7)	0.50
H5	0.9371	0.4481	0.0578	0.065*	0.50
N1A	−0.0147 (7)	0.7395 (6)	0.2448 (5)	0.0267 (12)	0.695 (5)
C1A	0.0554 (4)	0.7375 (4)	0.4310 (3)	0.0239 (6)	0.695 (5)
H1AA	0.0530	0.7777	0.4961	0.029*	0.695 (5)
C2A	−0.0181 (6)	0.7948 (5)	0.3438 (4)	0.0228 (8)	0.695 (5)
H2AA	−0.0707	0.8720	0.3517	0.027*	0.695 (5)
C3A	0.0609 (9)	0.6259 (8)	0.2357 (7)	0.0367 (15)	0.695 (5)
H3AA	0.0643	0.5885	0.1688	0.044*	0.695 (5)
C4A	0.1331 (4)	0.5640 (3)	0.3233 (4)	0.0332 (8)	0.695 (5)
H4AA	0.1818	0.4845	0.3157	0.040*	0.695 (5)
C5A	0.1341 (3)	0.6195 (3)	0.4234 (3)	0.0237 (6)	0.695 (5)
C6A	0.2090 (3)	0.5626 (3)	0.5195 (2)	0.0270 (7)	0.695 (5)
H6AA	0.1987	0.6051	0.5841	0.032*	0.695 (5)
C7A	0.2907 (3)	0.4544 (3)	0.5214 (2)	0.0261 (7)	0.695 (5)
H7AA	0.3008	0.4130	0.4561	0.031*	0.695 (5)
C8A	0.3662 (3)	0.3946 (3)	0.6161 (3)	0.0243 (6)	0.695 (5)
C9A	0.4440 (3)	0.2777 (4)	0.6085 (3)	0.0283 (7)	0.695 (5)
H9AA	0.4482	0.2397	0.5418	0.034*	0.695 (5)
C10A	0.5148 (6)	0.2167 (6)	0.6964 (5)	0.0294 (10)	0.695 (5)
H10A	0.5643	0.1378	0.6884	0.035*	0.695 (5)
C11A	0.5143 (8)	0.2692 (7)	0.7955 (6)	0.0287 (13)	0.695 (5)
C12A	0.4362 (6)	0.3892 (5)	0.8051 (5)	0.0348 (11)	0.695 (5)
H12A	0.4333	0.4268	0.8718	0.042*	0.695 (5)
C13A	0.3649 (3)	0.4505 (3)	0.7172 (4)	0.0302 (7)	0.695 (5)
H13A	0.3154	0.5295	0.7247	0.036*	0.695 (5)
C14A	−0.0929 (6)	0.8055 (6)	0.1557 (5)	0.0371 (11)	0.695 (5)
H14A	−0.0579	0.7792	0.0800	0.056*	0.695 (5)
H14B	−0.0841	0.9041	0.1414	0.056*	0.695 (5)
H14C	−0.1885	0.7771	0.1864	0.056*	0.695 (5)
C15A	0.6589 (7)	0.0983 (6)	0.8757 (7)	0.0231 (17)	0.695 (5)
H15A	0.7289	0.1197	0.7994	0.028*	0.695 (5)
H15B	0.5994	0.0236	0.8743	0.028*	0.695 (5)
C16A	0.7260 (12)	0.0566 (17)	0.9834 (10)	0.034 (2)	0.695 (5)
H16A	0.7892	−0.0160	0.9718	0.051*	0.695 (5)
H16B	0.6561	0.0243	1.0573	0.051*	0.695 (5)
H16C	0.7753	0.1349	0.9897	0.051*	0.695 (5)
N1B	−0.0172 (14)	0.7490 (16)	0.2597 (13)	0.020 (2)*	0.305 (5)
C1B	0.0848 (8)	0.7018 (9)	0.4259 (7)	0.0201 (18)*	0.305 (5)
H1BA	0.0962	0.7236	0.4971	0.024*	0.305 (5)
C2B	0.0053 (12)	0.7800 (14)	0.3562 (12)	0.024 (3)*	0.305 (5)
H2BA	−0.0344	0.8582	0.3782	0.029*	0.305 (5)
C3B	0.0405 (19)	0.638 (2)	0.2234 (19)	0.029 (3)*	0.305 (5)
H3BA	0.0207	0.6149	0.1556	0.035*	0.305 (5)
C4B	0.1273 (10)	0.5607 (11)	0.2859 (9)	0.030 (2)*	0.305 (5)
H4BA	0.1720	0.4881	0.2574	0.036*	0.305 (5)
C5B	0.1499 (7)	0.5881 (8)	0.3903 (8)	0.0212 (15)*	0.305 (5)
C6B	0.2386 (6)	0.5015 (6)	0.4641 (5)	0.0226 (14)*	0.305 (5)
H6BA	0.2810	0.4282	0.4351	0.027*	0.305 (5)
C7B	0.2637 (6)	0.5186 (6)	0.5688 (5)	0.0211 (14)*	0.305 (5)
H7BA	0.2213	0.5926	0.5966	0.025*	0.305 (5)
C8B	0.3503 (7)	0.4341 (8)	0.6447 (7)	0.0210 (14)*	0.305 (5)
C9B	0.4213 (8)	0.3225 (9)	0.6190 (7)	0.0241 (16)*	0.305 (5)
H9BA	0.4174	0.2964	0.5477	0.029*	0.305 (5)
C10B	0.5018 (16)	0.2447 (14)	0.6990 (15)	0.035 (4)*	0.305 (5)
H10B	0.5512	0.1691	0.6808	0.042*	0.305 (5)
C11B	0.5034 (16)	0.2863 (15)	0.8051 (13)	0.018 (3)*	0.305 (5)
C12B	0.4343 (11)	0.3971 (12)	0.8312 (10)	0.021 (2)*	0.305 (5)
H12B	0.4399	0.4246	0.9014	0.026*	0.305 (5)
C13B	0.3546 (9)	0.4703 (9)	0.7531 (8)	0.030 (2)*	0.305 (5)
H13B	0.3037	0.5442	0.7734	0.036*	0.305 (5)
C14B	−0.1068 (15)	0.8318 (13)	0.1724 (13)	0.028 (3)*	0.305 (5)
H14D	−0.1681	0.7690	0.1567	0.042*	0.305 (5)
H14E	−0.0475	0.8797	0.0963	0.042*	0.305 (5)
H14F	−0.1601	0.8975	0.2106	0.042*	0.305 (5)
C15B	0.659 (3)	0.098 (2)	0.877 (3)	0.054 (8)*	0.305 (5)
H15C	0.7338	0.1217	0.8046	0.065*	0.305 (5)
H15D	0.6004	0.0271	0.8673	0.065*	0.305 (5)
C16B	0.716 (3)	0.044 (4)	0.990 (3)	0.047 (9)*	0.305 (5)
H16D	0.7610	−0.0419	0.9852	0.071*	0.305 (5)
H16E	0.6426	0.0291	1.0619	0.071*	0.305 (5)
H16F	0.7825	0.1101	0.9937	0.071*	0.305 (5)
C17	0.42651 (18)	0.16977 (18)	0.29296 (16)	0.0244 (3)	
C18	0.39864 (19)	0.25307 (19)	0.18576 (17)	0.0288 (4)	
H18A	0.4697	0.3034	0.1249	0.035*	
C19	0.26369 (19)	0.26051 (19)	0.17041 (17)	0.0285 (4)	
H19A	0.2439	0.3161	0.0990	0.034*	
C20	0.15803 (18)	0.18491 (17)	0.26178 (16)	0.0233 (3)	
C21	0.18730 (19)	0.10245 (18)	0.36949 (16)	0.0252 (3)	
H21A	0.1163	0.0524	0.4307	0.030*	
C22	0.32179 (19)	0.09490 (18)	0.38552 (16)	0.0262 (3)	
H22A	0.3416	0.0404	0.4573	0.031*	
C23	0.7489 (5)	0.4042 (5)	0.0126 (4)	0.0433 (10)	0.50
H23A	0.7764	0.3470	−0.0440	0.065*	0.50
H23B	0.6475	0.4335	−0.0202	0.065*	0.50
H23C	0.7118	0.3469	0.0933	0.065*	0.50

### Atomic displacement parameters (Å^2^)

**Table:**

	*U*^11^	*U*^22^	*U*^33^	*U*^12^	*U*^13^	*U*^23^
Br1	0.02518 (9)	0.02959 (10)	0.03098 (10)	−0.00350 (7)	−0.00640 (7)	−0.00585 (7)
S1	0.0239 (2)	0.02263 (19)	0.0257 (2)	−0.00041 (15)	−0.00211 (16)	−0.00470 (16)
O1	0.0433 (8)	0.0253 (6)	0.0284 (7)	0.0050 (6)	−0.0099 (6)	−0.0086 (5)
O2	0.0247 (6)	0.0336 (7)	0.0307 (7)	−0.0064 (5)	−0.0021 (5)	−0.0010 (5)
O3	0.0341 (8)	0.0242 (7)	0.0566 (10)	0.0035 (6)	−0.0008 (7)	−0.0009 (6)
O4	0.0318 (7)	0.0584 (10)	0.0357 (8)	0.0039 (7)	−0.0091 (6)	−0.0239 (7)
O5	0.0519 (19)	0.0435 (17)	0.0314 (15)	0.0016 (14)	−0.0150 (14)	0.0030 (13)
N1A	0.044 (2)	0.0231 (18)	0.0148 (18)	−0.0134 (11)	−0.0077 (13)	−0.0057 (12)
C1A	0.0287 (14)	0.0213 (14)	0.0223 (14)	0.0016 (13)	−0.0060 (11)	−0.0064 (11)
C2A	0.0265 (19)	0.0225 (17)	0.0199 (17)	−0.0026 (14)	−0.0077 (14)	−0.0036 (12)
C3A	0.060 (4)	0.022 (2)	0.032 (2)	−0.004 (2)	−0.011 (3)	−0.0119 (18)
C4A	0.052 (2)	0.0206 (13)	0.0271 (18)	0.0008 (11)	−0.0114 (16)	−0.0042 (13)
C5A	0.0283 (13)	0.0190 (12)	0.0216 (14)	−0.0059 (10)	−0.0012 (10)	−0.0043 (11)
C6A	0.0322 (14)	0.0231 (12)	0.0256 (13)	−0.0034 (10)	−0.0039 (10)	−0.0079 (10)
C7A	0.0302 (13)	0.0231 (12)	0.0231 (12)	−0.0060 (10)	−0.0001 (10)	−0.0067 (10)
C8A	0.0284 (13)	0.0187 (13)	0.0230 (13)	−0.0032 (11)	0.0006 (10)	−0.0051 (11)
C9A	0.0327 (15)	0.0245 (15)	0.0274 (14)	0.0011 (12)	−0.0010 (11)	−0.0110 (12)
C10A	0.0321 (19)	0.026 (2)	0.0288 (18)	0.0007 (16)	−0.0019 (12)	−0.0084 (16)
C11A	0.034 (2)	0.018 (2)	0.030 (2)	0.0008 (14)	−0.0010 (16)	−0.0034 (15)
C12A	0.054 (2)	0.0253 (17)	0.028 (2)	−0.0017 (12)	−0.0086 (18)	−0.0111 (16)
C13A	0.0373 (16)	0.0173 (13)	0.0323 (19)	0.0016 (10)	−0.0011 (14)	−0.0057 (13)
C14A	0.045 (3)	0.033 (3)	0.030 (2)	−0.012 (2)	−0.0155 (19)	0.0052 (18)
C15A	0.0195 (18)	0.0215 (19)	0.026 (2)	0.0024 (9)	−0.0013 (9)	−0.0062 (10)
C16A	0.025 (2)	0.040 (3)	0.032 (3)	0.002 (2)	−0.0059 (15)	0.0003 (18)
C17	0.0246 (8)	0.0231 (8)	0.0259 (8)	−0.0016 (6)	−0.0043 (6)	−0.0078 (6)
C18	0.0267 (8)	0.0268 (8)	0.0277 (9)	−0.0070 (7)	−0.0015 (7)	−0.0005 (7)
C19	0.0298 (9)	0.0262 (8)	0.0255 (8)	−0.0041 (7)	−0.0046 (7)	0.0001 (7)
C20	0.0234 (8)	0.0204 (7)	0.0255 (8)	−0.0028 (6)	−0.0018 (6)	−0.0074 (6)
C21	0.0264 (8)	0.0243 (8)	0.0226 (8)	−0.0034 (6)	0.0000 (6)	−0.0060 (6)
C22	0.0294 (9)	0.0249 (8)	0.0231 (8)	−0.0024 (7)	−0.0040 (7)	−0.0050 (6)
C23	0.057 (3)	0.033 (2)	0.032 (2)	0.0013 (19)	0.0035 (19)	−0.0064 (17)

### Geometric parameters (Å, °)

**Table:**

Br1—C17	1.8962 (18)	N1B—C3B	1.35 (2)
S1—O4	1.4420 (15)	N1B—C14B	1.56 (2)
S1—O2	1.4575 (14)	C1B—C2B	1.349 (12)
S1—O3	1.4600 (14)	C1B—C5B	1.394 (10)
S1—C20	1.7780 (18)	C1B—H1BA	0.9300
O1—C11A	1.366 (5)	C2B—H2BA	0.9300
O1—C11B	1.394 (8)	C3B—C4B	1.351 (17)
O1—C15B	1.434 (9)	C3B—H3BA	0.9300
O1—C15A	1.436 (3)	C4B—C5B	1.364 (10)
O5—C23	1.716 (6)	C4B—H4BA	0.9300
O5—H5	0.8200	C5B—C6B	1.476 (9)
N1A—C3A	1.355 (10)	C6B—C7B	1.336 (8)
N1A—C2A	1.379 (6)	C6B—H6BA	0.9300
N1A—C14A	1.445 (8)	C7B—C8B	1.467 (9)
C1A—C2A	1.376 (6)	C7B—H7BA	0.9300
C1A—C5A	1.405 (5)	C8B—C9B	1.360 (10)
C1A—H1AA	0.9300	C8B—C13B	1.394 (10)
C2A—H2AA	0.9300	C9B—C10B	1.427 (17)
C3A—C4A	1.381 (8)	C9B—H9BA	0.9300
C3A—H3AA	0.9300	C10B—C11B	1.39 (2)
C4A—C5A	1.400 (5)	C10B—H10B	0.9300
C4A—H4AA	0.9300	C11B—C12B	1.346 (17)
C5A—C6A	1.463 (4)	C12B—C13B	1.391 (14)
C6A—C7A	1.331 (4)	C12B—H12B	0.9300
C6A—H6AA	0.9300	C13B—H13B	0.9300
C7A—C8A	1.460 (4)	C14B—H14D	0.9600
C7A—H7AA	0.9300	C14B—H14E	0.9600
C8A—C9A	1.391 (4)	C14B—H14F	0.9600
C8A—C13A	1.408 (5)	C15B—C16B	1.511 (10)
C9A—C10A	1.373 (7)	C15B—H15C	0.9700
C9A—H9AA	0.9300	C15B—H15D	0.9700
C10A—C11A	1.368 (8)	C16B—H16D	0.9600
C10A—H10A	0.9300	C16B—H16E	0.9600
C11A—C12A	1.422 (8)	C16B—H16F	0.9600
C12A—C13A	1.377 (6)	C17—C22	1.388 (2)
C12A—H12A	0.9300	C17—C18	1.389 (3)
C13A—H13A	0.9300	C18—C19	1.388 (3)
C14A—H14A	0.9600	C18—H18A	0.9300
C14A—H14B	0.9600	C19—C20	1.390 (2)
C14A—H14C	0.9600	C19—H19A	0.9300
C15A—C16A	1.503 (5)	C20—C21	1.396 (2)
C15A—H15A	0.9700	C21—C22	1.386 (3)
C15A—H15B	0.9700	C21—H21A	0.9300
C16A—H16A	0.9600	C22—H22A	0.9300
C16A—H16B	0.9600	C23—H23A	0.9600
C16A—H16C	0.9600	C23—H23B	1.1586
N1B—C2B	1.291 (17)	C23—H23C	0.9600
			
O4—S1—O2	113.62 (9)	C3B—C4B—H4BA	119.7
O4—S1—O3	113.59 (10)	C5B—C4B—H4BA	119.7
O2—S1—O3	112.28 (9)	C4B—C5B—C1B	117.3 (8)
O4—S1—C20	105.52 (8)	C4B—C5B—C6B	122.1 (8)
O2—S1—C20	105.47 (8)	C1B—C5B—C6B	120.6 (8)
O3—S1—C20	105.41 (9)	C7B—C6B—C5B	126.1 (6)
C11A—O1—C15B	114.3 (11)	C7B—C6B—H6BA	116.9
C11B—O1—C15B	124.0 (12)	C5B—C6B—H6BA	116.9
C11A—O1—C15A	113.7 (4)	C6B—C7B—C8B	127.5 (6)
C11B—O1—C15A	123.5 (7)	C6B—C7B—H7BA	116.2
C23—O5—H5	109.5	C8B—C7B—H7BA	116.2
C3A—N1A—C2A	118.7 (6)	C9B—C8B—C13B	118.8 (7)
C3A—N1A—C14A	123.1 (6)	C9B—C8B—C7B	125.0 (7)
C2A—N1A—C14A	118.2 (6)	C13B—C8B—C7B	116.2 (7)
C2A—C1A—C5A	121.2 (3)	C8B—C9B—C10B	121.4 (9)
C2A—C1A—H1AA	119.4	C8B—C9B—H9BA	119.3
C5A—C1A—H1AA	119.4	C10B—C9B—H9BA	119.3
C1A—C2A—N1A	120.9 (5)	C11B—C10B—C9B	117.4 (10)
C1A—C2A—H2AA	119.5	C11B—C10B—H10B	121.3
N1A—C2A—H2AA	119.5	C9B—C10B—H10B	121.3
N1A—C3A—C4A	121.8 (7)	C12B—C11B—C10B	121.8 (9)
N1A—C3A—H3AA	119.1	C12B—C11B—O1	116.1 (10)
C4A—C3A—H3AA	119.1	C10B—C11B—O1	122.1 (11)
C3A—C4A—C5A	120.9 (4)	C11B—C12B—C13B	120.0 (9)
C3A—C4A—H4AA	119.6	C11B—C12B—H12B	120.0
C5A—C4A—H4AA	119.6	C13B—C12B—H12B	120.0
C4A—C5A—C1A	116.6 (3)	C12B—C13B—C8B	120.6 (8)
C4A—C5A—C6A	124.8 (3)	C12B—C13B—H13B	119.7
C1A—C5A—C6A	118.7 (3)	C8B—C13B—H13B	119.7
C7A—C6A—C5A	124.9 (3)	N1B—C14B—H14D	109.5
C7A—C6A—H6AA	117.6	N1B—C14B—H14E	109.5
C5A—C6A—H6AA	117.6	H14D—C14B—H14E	109.5
C6A—C7A—C8A	126.2 (3)	N1B—C14B—H14F	109.5
C6A—C7A—H7AA	116.9	H14D—C14B—H14F	109.5
C8A—C7A—H7AA	116.9	H14E—C14B—H14F	109.5
C9A—C8A—C13A	117.2 (3)	O1—C15B—C16B	110 (2)
C9A—C8A—C7A	120.0 (3)	O1—C15B—H15C	109.7
C13A—C8A—C7A	122.8 (3)	C16B—C15B—H15C	109.7
C10A—C9A—C8A	122.0 (4)	O1—C15B—H15D	109.7
C10A—C9A—H9AA	119.0	C16B—C15B—H15D	109.7
C8A—C9A—H9AA	119.0	H15C—C15B—H15D	108.2
C11A—C10A—C9A	121.6 (5)	C15B—C16B—H16D	109.5
C11A—C10A—H10A	119.2	C15B—C16B—H16E	109.5
C9A—C10A—H10A	119.2	H16D—C16B—H16E	109.5
O1—C11A—C10A	127.3 (5)	C15B—C16B—H16F	109.5
O1—C11A—C12A	115.1 (5)	H16D—C16B—H16F	109.5
C10A—C11A—C12A	117.5 (4)	H16E—C16B—H16F	109.5
C13A—C12A—C11A	121.0 (4)	C22—C17—C18	121.26 (17)
C13A—C12A—H12A	119.5	C22—C17—Br1	119.00 (13)
C11A—C12A—H12A	119.5	C18—C17—Br1	119.71 (13)
C12A—C13A—C8A	120.7 (3)	C19—C18—C17	119.35 (16)
C12A—C13A—H13A	119.7	C19—C18—H18A	120.3
C8A—C13A—H13A	119.7	C17—C18—H18A	120.3
O1—C15A—C16A	107.4 (7)	C18—C19—C20	120.00 (17)
O1—C15A—H15A	110.2	C18—C19—H19A	120.0
C16A—C15A—H15A	110.2	C20—C19—H19A	120.0
O1—C15A—H15B	110.2	C19—C20—C21	120.06 (16)
C16A—C15A—H15B	110.2	C19—C20—S1	119.50 (14)
H15A—C15A—H15B	108.5	C21—C20—S1	120.42 (13)
C2B—N1B—C3B	121.0 (14)	C22—C21—C20	120.21 (16)
C2B—N1B—C14B	126.1 (12)	C22—C21—H21A	119.9
C3B—N1B—C14B	112.9 (13)	C20—C21—H21A	119.9
C2B—C1B—C5B	119.4 (9)	C21—C22—C17	119.11 (16)
C2B—C1B—H1BA	120.3	C21—C22—H22A	120.4
C5B—C1B—H1BA	120.3	C17—C22—H22A	120.4
N1B—C2B—C1B	121.7 (12)	O5—C23—H23A	109.3
N1B—C2B—H2BA	119.1	O5—C23—H23B	133.8
C1B—C2B—H2BA	119.1	H23A—C23—H23B	92.4
N1B—C3B—C4B	119.7 (18)	O5—C23—H23C	109.5
N1B—C3B—H3BA	120.1	H23A—C23—H23C	109.5
C4B—C3B—H3BA	120.1	H23B—C23—H23C	100.1
C3B—C4B—C5B	120.7 (13)		
			
C5A—C1A—C2A—N1A	−1.2 (6)	C2B—C1B—C5B—C4B	−0.9 (12)
C3A—N1A—C2A—C1A	1.0 (9)	C2B—C1B—C5B—C6B	179.4 (8)
C14A—N1A—C2A—C1A	−179.3 (4)	C4B—C5B—C6B—C7B	−178.3 (7)
C2A—N1A—C3A—C4A	0.6 (11)	C1B—C5B—C6B—C7B	1.4 (10)
C14A—N1A—C3A—C4A	−179.2 (6)	C5B—C6B—C7B—C8B	179.5 (6)
N1A—C3A—C4A—C5A	−2.0 (10)	C6B—C7B—C8B—C9B	0.5 (11)
C3A—C4A—C5A—C1A	1.6 (6)	C6B—C7B—C8B—C13B	−177.2 (7)
C3A—C4A—C5A—C6A	−179.0 (5)	C13B—C8B—C9B—C10B	−1.4 (13)
C2A—C1A—C5A—C4A	−0.1 (5)	C7B—C8B—C9B—C10B	−179.1 (9)
C2A—C1A—C5A—C6A	−179.5 (3)	C8B—C9B—C10B—C11B	0.8 (19)
C4A—C5A—C6A—C7A	3.1 (4)	C9B—C10B—C11B—C12B	−1(2)
C1A—C5A—C6A—C7A	−177.6 (3)	C9B—C10B—C11B—O1	−179.6 (12)
C5A—C6A—C7A—C8A	−179.6 (2)	C11A—O1—C11B—C12B	−176 (8)
C6A—C7A—C8A—C9A	177.5 (3)	C15B—O1—C11B—C12B	179.7 (18)
C6A—C7A—C8A—C13A	−2.7 (4)	C15A—O1—C11B—C12B	180.0 (10)
C13A—C8A—C9A—C10A	1.2 (5)	C11A—O1—C11B—C10B	2(5)
C7A—C8A—C9A—C10A	−179.0 (3)	C15B—O1—C11B—C10B	−2(3)
C8A—C9A—C10A—C11A	−1.0 (7)	C15A—O1—C11B—C10B	−2(2)
C11B—O1—C11A—C10A	−175 (7)	C10B—C11B—C12B—C13B	2(2)
C15B—O1—C11A—C10A	1.7 (17)	O1—C11B—C12B—C13B	−179.3 (11)
C15A—O1—C11A—C10A	1.9 (10)	C11B—C12B—C13B—C8B	−2.9 (17)
C11B—O1—C11A—C12A	4(6)	C9B—C8B—C13B—C12B	2.4 (13)
C15B—O1—C11A—C12A	−179.4 (15)	C7B—C8B—C13B—C12B	−179.6 (8)
C15A—O1—C11A—C12A	−179.1 (6)	C11A—O1—C15B—C16B	−176 (2)
C9A—C10A—C11A—O1	179.6 (5)	C11B—O1—C15B—C16B	−175 (2)
C9A—C10A—C11A—C12A	0.6 (9)	C15A—O1—C15B—C16B	158 (100)
O1—C11A—C12A—C13A	−179.6 (5)	C22—C17—C18—C19	−0.6 (3)
C10A—C11A—C12A—C13A	−0.5 (9)	Br1—C17—C18—C19	177.40 (14)
C11A—C12A—C13A—C8A	0.8 (7)	C17—C18—C19—C20	−0.1 (3)
C9A—C8A—C13A—C12A	−1.1 (5)	C18—C19—C20—C21	0.6 (3)
C7A—C8A—C13A—C12A	179.1 (3)	C18—C19—C20—S1	−178.10 (14)
C11A—O1—C15A—C16A	178.8 (8)	O4—S1—C20—C19	62.92 (17)
C11B—O1—C15A—C16A	179.5 (11)	O2—S1—C20—C19	−176.53 (14)
C15B—O1—C15A—C16A	−27 (100)	O3—S1—C20—C19	−57.57 (17)
C3B—N1B—C2B—C1B	−1(2)	O4—S1—C20—C21	−115.77 (15)
C14B—N1B—C2B—C1B	−179.9 (12)	O2—S1—C20—C21	4.79 (16)
C5B—C1B—C2B—N1B	3.0 (18)	O3—S1—C20—C21	123.75 (15)
C2B—N1B—C3B—C4B	−2(3)	C19—C20—C21—C22	−0.4 (3)
C14B—N1B—C3B—C4B	176.3 (14)	S1—C20—C21—C22	178.28 (13)
N1B—C3B—C4B—C5B	4(2)	C20—C21—C22—C17	−0.3 (3)
C3B—C4B—C5B—C1B	−2.7 (16)	C18—C17—C22—C21	0.8 (3)
C3B—C4B—C5B—C6B	176.9 (12)	Br1—C17—C22—C21	−177.23 (13)

### Hydrogen-bond geometry (Å, °)

**Table:**

*Cg*1, *Cg*2, *Cg*3, *Cg*4 and *Cg*5 are the centroids of the N1A/C1A–C5A, C8A–C13A, N1B/C1B–C5B, C8B–C13B and C17–C22 rings, respectively.

**Table:**

*D*—H···*A*	*D*—H	H···*A*	*D*···*A*	*D*—H···*A*
O5—H5···O3^i^	0.82	1.92	2.657 (4)	149
C1A—H1AA···O2^ii^	0.93	2.42	3.334 (4)	168
C2A—H2AA···O2^iii^	0.93	2.38	3.237 (5)	153
C14A—H14A···O4^iv^	0.96	2.36	3.180 (6)	143
C14A—H14B···O4^iii^	0.96	2.41	3.343 (6)	163
C19—H19A···O5^v^	0.93	2.53	3.385 (4)	153
C21—H21A···O2^vi^	0.93	2.58	3.311 (2)	135
C23—H23C···Br1	0.96	2.77	3.724 (5)	173
C14A—H14C···Cg2^ii^	0.96	2.66	3.609 (6)	172
C14A—H14C···Cg4^ii^	0.96	2.63	3.572 (8)	167
C15A—H15A···Cg1^vii^	0.97	2.84	3.639 (8)	140
C15A—H15A···Cg3^vii^	0.97	2.84	3.611 (9)	137
C14B—H14D···Cg2^ii^	0.96	2.87	3.562 (15)	129
C14B—H14D···Cg4^ii^	0.96	2.80	3.564 (16)	137
C15B—H15C···Cg1^vii^	0.97	2.81	3.65 (3)	145
C15B—H15C···Cg3^vii^	0.97	2.81	3.62 (3)	141
C15B—H15D···Cg5^viii^	0.97	2.98	3.60 (2)	123

Symmetry codes: (i) *x*+1, *y*, *z*; (ii) −*x*, −*y*+1, −*z*+1; (iii) *x*, *y*+1, *z*; (iv) −*x*, −*y*+1, −*z*; (v) −*x*+1, −*y*+1, −*z*; (vi) −*x*, −*y*, −*z*+1;  (vii) −*x*+1, −*y*+1, −*z*+1; (viii) −*x*+1, −*y*, −*z*+1.
